# Effects of a Long-Term Supervised Schroth Exercise Program on the Severity of Scoliosis and Quality of Life in Individuals with Adolescent Idiopathic Scoliosis: A Randomized Clinical Trial Study

**DOI:** 10.3390/medicina60101637

**Published:** 2024-10-07

**Authors:** Athanasios Kyrkousis, Paris Iakovidis, Ioanna P. Chatziprodromidou, Dimitrios Lytras, Konstantinos Kasimis, Thomas Apostolou, Georgios Koutras

**Affiliations:** 1 Department of Physiotherapy, Faculty of Health Sciences, International Hellenic University, Alexander Campus, Sindos, 57400 Thessaloniki, Greece; sakis.kirkousis@gmail.com (A.K.); piakov@ihu.gr (P.I.); ioannachatzi@med.upatras.gr (I.P.C.); konstantinoskasimis@gmail.com (K.K.); apostolouthomas@ihu.gr (T.A.); kutrasg@otenet.gr (G.K.); 2 Laboratory of Biomechanics & Ergonomics, Department of Physiotherapy, Faculty of Health Sciences, International Hellenic University, Alexander Campus, Sindos, 57400 Thessaloniki, Greece; 3 Department of Public Health, Medical School, University of Patras, 26504 Patras, Greece

**Keywords:** adolescent idiopathic scoliosis, Schroth exercises, conservative treatment, physical therapy

## Abstract

*Background and Objectives*: Adolescent Idiopathic Scoliosis (AIS) affects individuals aged 10–18 years and is characterized by spinal deformity, three-dimensional axis deformation, and vertebral rotation. Schroth method exercises and braces have been shown to reduce the Cobb angle and halt spinal deformity progression. The aim of this study was to investigate the impact of a 12-month, supervised Schroth exercise program on scoliosis severity and quality of life in adolescents with AIS. *Materials and Methods*: Eighty adolescents with AIS (aged 10–17 years) were prescribed a brace and were divided into two groups. The intervention group followed a supervised Schroth exercise program three times a week for 12 months in addition to wearing a brace. The control group used only the brace. Outcomes included the Cobb angle of the main curvature and the sum of curves using radiography, the maximum angle of trunk rotation (ATR maximum, using a scoliometer), and quality of life with the Scoliosis Research Society-22 (SRS-22) questionnaire. Evaluations were conducted at baseline, after 12 months, and 6 months post-intervention. A multivariate analysis of covariance (MANCOVA) was used for statistical analysis (*p*-Value < 0.05). *Results*: The intervention group showed statistically significant improvement compared to the control group in the 12th month in Cobb angle (mean differences, 95% CI: −3.65 (−5.81, −1.53), *p*-Value < 0.001, Cohen’s d = 0.30), ATR maximum (mean differences, 95% CI: −3.05 (−3.86, −2.23), *p*-Value < 0.001, Cohen’s d = 0.74), and SRS-22 score (mean differences, 95% CI: 0.87 (0.60, 1.13), *p*-Value < 0.001, Cohen’s d = 0.58). Differences in ATR maximum and SRS-22 score remained significant at the 18-month measurement. No significant differences were found between groups in the sum of curves (*p*-Value > 0.05). *Conclusions*: A 12-month supervised Schroth exercise program in AIS patients undergoing brace treatment significantly improves scoliosis severity (Cobb angle and ATR maximum) and quality of life. Improvements were greater than those in shorter-duration studies, suggesting a linear dose–response relationship. Further clinical studies are needed to clarify the impact of long-term Schroth programs.

## 1. Introduction

Idiopathic scoliosis is a condition that affects the spine and is characterized by ab-normal curvature growth. It involves three-dimensional deformation and potential vertebral rotation [1]. Adolescent Idiopathic Scoliosis (AIS) is the most common form of idiopathic scoliosis, accounting for up to 90% of cases among individuals aged 10–18 years [2,3,4]. The International Society on Scoliosis Orthopaedic and Rehabilitation Treatment (SOSORT) estimates that AIS affects up to 12% of children and adolescents worldwide [5].

The etiopathogenesis of AIS remains unknown, although various factors such as heredity and metabolic disorders (primarily melatonin metabolism) have been implicated [6]. The condition occurs more frequently in girls, and typical manifestations may include noticeable spinal and thoracic deformities, which can compromise lung function, cause breathing difficulties, impair posture, and restrict body movement. These changes often lead to discomfort, spinal pain syndromes, and reduced flexibility, while also negatively impacting an individual’s self-image, confidence, and overall quality of life. In more severe cases, the visible curvature of the spine can contribute to social anxiety and emotional distress, further intensifying the psychological burden of the condition [7,8].

The management of AIS depends on the patient’s skeletal maturity, deformity magnitude, and curve progression. Therefore, treatment must be individualized [5]. Various interventions for AIS are currently available, and they are typically classified into conservative and surgical treatments [5,9]. While surgical interventions seem to be the recommended approach for severe scoliosis, conservative treatment is primarily recommended for patients with a mild to moderate curvature [9]. Conservative management includes daily use of a brace for at least 16 h a day, as well as the implementation of therapeutic exercises [5,10]. Targeted exercises are a fundamental aspect of conservative treatment [10]. The International Society on Scoliosis Orthopaedic and Rehabilitation Treatment (SOSORT) refers to these as “Physiotherapeutic scoliosis-specific exercises” (PSSEs), encompassing various approaches from different countries [3]. Among the different PSSE approaches, the Schroth method appears to be the most effective and widely used in clinical practice [3]. The Schroth method utilizes 3D autocorrection, self-elongation, corrective breathing, and training for activities of daily living, while employing a classification system with “Body Blocks” to illustrate trunk deformation [9,10,11,12].

Recent studies suggest that Schroth exercises may be associated with positive effects on the severity of scoliosis and quality of life in individuals with AIS [13,14,15]. Although a recent meta-analysis suggests that Schroth programs longer than 6 months seem to be more effective than those shorter than 6 months [14], few studies implement programs lasting more than 6 months [10,14,15]. Furthermore, even in cases where the duration of Schroth exercise programs exceeds 6 months, the exercises are often given as home instructions after a few weeks of supervision rather than being conducted under continuous supervision by a physiotherapist [16,17]. However, Kwan et al. [16] found that a supervised Schroth exercise program resulted in statistically significant improvements in scoliosis severity parameters compared to both a home exercise group and a control group.

The purpose of our study was to examine the effects of a 12-month face-to-face supervised Schroth exercise program on the severity of scoliosis and quality of life in adolescents with AIS who are also undergoing brace treatment. Our research was based on the hypothesis that adding a long-term Schroth exercise program to brace treatment could significantly improve parameters related to the severity of AIS and the quality of life in these adolescents.

## 2. Materials and Methods

### 2.1. Study Design

This clinical study was conducted under the supervision of the Department of Physiotherapy at the School of Health Sciences, International Hellenic University. The Ethics Committee of the Department of Physiotherapy at the International Hellenic University approved the conduct of this study (approval number: EC-9 2022), and the clinical study protocol was retrospectively registered with the protocol ID: NCT06500806.

**Figure 1:**
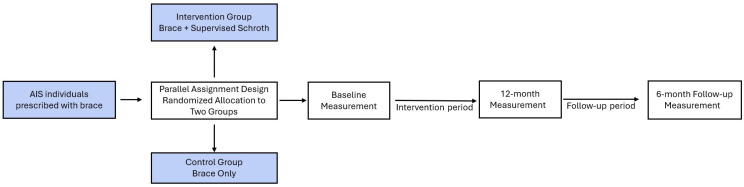
Graphical representation of the study design.

The sample included adolescents aged 10–17 years with mild to moderate scoliosis (Cobb angle ranging from 10° to 45°) who had been prescribed a brace. Participants were randomly assigned to two groups. The first group (intervention group) participated in a supervised Schroth exercise program for 12 months, with sessions three times a week, in addition to using a brace. The second group followed only the brace treatment and received recommendations for physical activity (Figure 1).

The intervention lasted for one year, with a follow-up six months after the end of the exercise program (18th month). The research team members provided written information to the parents and adolescents about the purpose of the study, and all parents provided written consent for their children’s participation.

### 2.2. Sampling, Randomization, and Blinding

The sampling of study participants was conducted using simple random sampling after an open invitation from the research team members to the patients of the two participating clinics. The sample collection period was two months (December 2022–January 2023). Individuals deemed eligible for the study were randomly assigned to two groups using randomization software. During randomization, gender matching was used to ensure an equal ratio of males to females in both groups. The randomization was conducted by a blinded member of the research team to maintain allocation concealment. Only the outcome assessors were blinded to the different groups. Neither the participants nor the treatment providers could be blinded to the aim of the study.

### 2.3. Participants

Participants had to meet the following eligibility criteria to be included in the study: (1) Diagnosed with AIS, (2) Aged 10–17 years and of either gender (the age range was based on the AIS guidelines of the International Scientific Society on Scoliosis Othopaedic and Rehabilitation Treatment (SOSORT) [5]), (3) Have a Cobb angle between 10–45°, (4) Have a Risser grade of 0–3, (5) Prescribed a scoliosis brace, (6) Have a signed written consent form from a legal guardian, and (7) Able to attend Schroth exercise sessions for one year. The exclusion criteria were: (1) Any contraindication for exercise, (2) Scheduled surgery for scoliosis, (3) Diagnosed mental disorders (e.g., intellectual disability, autism), (4) Diagnosed neurological or rheumatic diseases, (5) Non-idiopathic scoliosis, and (6) Previous corrective spinal surgery.

### 2.4. Measurements

The following measurements were taken at the beginning of the study, after 12 months (completion of the Schroth exercise program), and after 18 months (6-month follow-up).

#### 2.4.1. Cobb Angle

The Cobb angle was measured by an independent orthopedic evaluator using the Surgimap 2.3.2.1 software, (Nemaris, Inc., Surgimap®, Methuen, MA, USA). To assess the Cobb angle at each time point, the participants underwent a radiographic examination (plain X-ray) with an anteroposterior view of the entire spine in an upright position. In this study, both the Cobb angle of the primary (largest) curvature and the sum of the Cobb angles of all curvatures were recorded.

#### 2.4.2. Ankle Trunk Rotation, ATR

The ATR was measured using a scoliometer (Pedi-Scoliometer, Pedihealth, Finland). For the ATR measurement, each participant stood with feet together in an upright position and performed a forward bend (Adam’s forward bend test) until their back was parallel to the ground. The scoliometer was then placed vertically along the spine at the level of the rib hump. In this study, the maximum ATR (ATR maximum) was recorded.

#### 2.4.3. Quality of Life with the SRS-22 Questionnaire

The participants’ quality of life was assessed using the Scoliosis Research Society 22 (SRS-22) questionnaire [18]. The SRS-22 is the official tool of the Scoliosis Research Society for evaluating quality of life in individuals with scoliosis. It includes twenty-two questions divided into five subcategories: five questions on function, five on pain, five on self-image, five on mental health, and two on satisfaction with the treatment. Each response is rated on a Likert scale from 1 to 5. The total score of the questionnaire is calculated by averaging the scores of the five subcategories, ranging from 1 (low) to 5 (high), with a higher score indicating a better overall quality of life. For this study, the Greek version of the questionnaire was used [19]. The total SRS-22 score was used to determine the level of quality of life. The reliability and validity of the Greek version of the questionnaire have been found to be very high compared to the 36-Item Short Form Survey (SF-36) [19].

### 2.5. Experimental Protocols

#### 2.5.1. Brace and Schroth Exercise Group Protocol

The participants in the first group wore a brace and underwent a 12-month Schroth exercise program. Sessions were held three times a week. The protocol was based on previous similar studies [16,20]. Exercises were performed at the clinic under the supervision of a trained physiotherapist, with each session lasting 60 min. Special equipment such as foam blocks, exercise balls, and long rods were used for posture adjustment and support. Exercise intensity gradually increased based on individual progress. The Schroth exercise regimen was adapted for each participant, targeting their specific deformity patterns and aimed to achieve spinal correction through asymmetrical positioning. It included spinal elongation, rotational correction exercises, stretching, strengthening, and breathing exercises designed to maintain vertebral alignment. The goal of these exercises was to help patients consciously maintain proper posture in their daily activities, enhancing their postural control. In this study, Schroth exercises were adapted to the functional ability of each patient, following a graded difficulty approach in both support provision (from more passive to more active exercises) and exercise positions (ground, sitting, or standing).

#### 2.5.2. Brace Group Protocol

The participants in this group received instructions on the proper use of the brace and recommendations for engaging in physical activity without following a specific Schroth exercise regimen.

### 2.6. Sample Size Estimation

Determining the sample size for the study involved utilizing the G*Power software, version 3.0.10 (Franz Faul, University of Kiel, Germany). The estimation was guided by key parameters: a study power (1-β) of 80%, a Type I error rate (α) of 0.05, and an effect size (f) of 0.4, referencing Cohen [21]. The effect size of 0.4 was considered acceptable based on the number of participants used in similar studies [20,22], in order to enhance the practical feasibility of the study [23]. The calculated minimum number of participants required was 64. To accommodate potential data loss over the 6-month follow-up period, an additional 20% was included, leading to a final minimum sample size of 80 participants.

### 2.7. Statistical Analysis

The statistical analysis of the data was conducted using the Statistical Package for the Social Sciences (SPSS) software, version 25.0 (SPSS Inc., Chicago, IL, USA). Descriptive analysis and frequency analysis were performed to present the demographic characteristics of the participants. The normality of the distribution of quantitative variables was checked using the Shapiro-Wilk test and Q-Q plots. The mean and standard deviations were reported for normally distributed values.

A multivariate analysis of covariance (MANCOVA) with repeated measures was used to examine the effect of the Schroth program on the primary outcomes of the study in Cobb angle (of the largest curvature and sum of curves), maximum ATR, and SRS-22 score. The “group” factor was examined at two levels (intervention group and control group) and the “time” factor at three levels (baseline measurement, 12-month measurement, and 18-month follow-up). The Risser index (0–3) was added as a covariate in the multivariate analysis model because it reflects skeletal maturity and indirectly indicates the potential for scoliosis progression, which could directly affect the intervention outcomes.

Before conducting the MANCOVA, all necessary assumptions for the analysis such as coherence, normality, homogeneity of variances, and sphericity of dependent variables were verified and met. Post-hoc analyses were performed to examine detailed interactions between the group and time using independent and dependent *t*-tests. The effect size for all post-hoc tests was calculated using Cohen’s d [21]. All effect sizes and the power of the analyses (both multivariate and univariate) were computed using G*Power 3.1. Effect sizes for Cohen’s d were categorized as small (d = 0.2), medium (d = 0.5), and large (d = 0.8) according to Cohen [21].

To manage missing data, an Intention to Treat (ITT) analysis was applied to preserve the randomized group allocation. All participants were included in the analysis and analyzed in their originally assigned groups. For each dropout during the intervention period, missing values were replaced with the last observed value of each variable. Statistical significance was set at the 0.05 level.

## 3. Results

**Figure 2:**
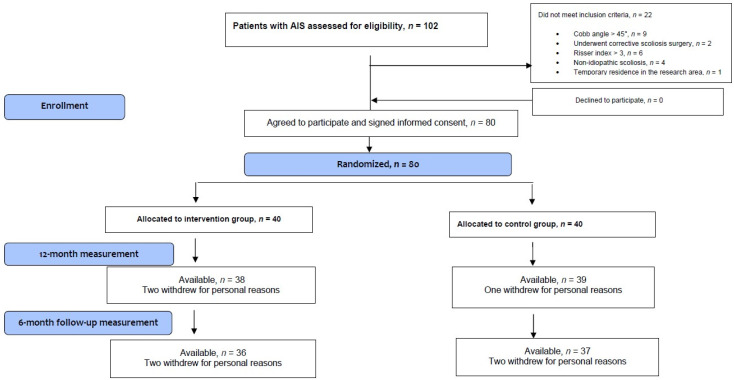
CONSORT flow diagram of the study.

During the participant recruitment period, a total of 102 adolescents with scoliosis were screened. Eighty (78.3%) were included in the study. The sample collection process ceased once this number was reached as it was minimum required sample size, according to the sample size calculation (see Section 2.6. Regarding the twenty-two individuals excluded, nine had a primary curvature with a Cobb angle greater than 45°, two had undergone corrective scoliosis surgery, six had a Risser index greater than three, four were diagnosed with a type of scoliosis other than idiopathic, and one was temporarily residing in the research area. The reasons for participant exclusion are detailed in the study flowchart (Figure 2). Furthermore, out of the eighty individuals that were randomized into two groups (forty in each group), three did not attend the 12-month measurement (two from the intervention group and one from the control group), and an additional four individuals (two from each group) stopped communication during the 6-month follow-up (Figure 2). No other exercise sessions or measurement evaluations were missed during the 18-month study period. If an exercise session was missed during the intervention period, an effort was made to reschedule it within the same week. In case of illness, each participant could miss the program for up to two weeks. No participant missed more than seven days due to illness during the exercise program. Additionally, no complications or adverse symptoms were reported by any participant during the intervention period.

Participants had a mean age of 13.72 (1.15) years, with 72% being female. The mean Cobb angle of the primary curvature was 34.04 (4.76) degrees, and the mean ATR maximum was 13.21 (2.32) degrees. The mean Risser index was 1.55 (0.99), the mean BMI was 19.80 (2.17) kg/m^2^, the mean age of menarche was 12.75 (0.99) years, and the mean daily brace wear time was 18.05 (0.22) hours. Additionally, 62.5% had no family history of scoliosis and 77.5% participated in some form of weekly sports activity.

Independent *t*-tests were applied to the quantitative variables between the means of the two groups to examine the differences between the groups in terms of their demographic characteristics and scoliosis severity (Cobb angle and ATR max of the primary curvature) and the chi-square test (χ^2^) was used for the categorical variables between the groups. The tests revealed no statistically significant differences between the groups. The demographic characteristics of the participants by group and the *p*-Values of the tests for examining group differences are presented in Table 1.

### 3.1. Multivariate Outcomes

The results of the repeated measures MANCOVA showed a statistically significant multivariate effect between the two groups (V = 0.566, F(74.00) = 14.20, *p*-Value < 0.001, effect size = 1.14, power = 1). The covariate introduced into the analysis model (Risser index) did not show a statistically significant effect (*p*-Value = 0.39). Additionally, there was a statistically significant multivariate effect of the time factor across the three different measurement points (baseline, 12 months, 18 months) (Λ = 0.09, F(70.00) = 80.71, *p*-Value < 0.001, effect size = 0.32, power = 0.99). No multivariate effect of the Risser index covariate was found across the different measurement times (*p*-Value = 0.49). Finally, there was a statistically significant multivariate interaction between the group and time factors (Λ = 0.82, F(70.00) = 42.28, *p*-Value < 0.001, effect size = 2.20, power = 1).

### 3.2. Univariate Outcomes

#### 3.2.1. Cobb Angle Results

The analysis results showed a statistically significant effect of the measurement time factor (F(1.78) = 25.1, *p*-Value < 0.001, effect size = 0.57, power = 1) and a statistically significant interaction between the time and group factors (F(1.31) = 149.75, *p*-Value < 0.001, effect size = 0.30, power = 0.99). Post-hoc tests revealed statistically significant differences between the groups at the 12-month measurement (t(52.87) = −3.44, *p*-Value = 0.001), with the intervention group showing a statistically significant improvement compared to the control group (mean difference −3.67). This difference was not statistically significant at the 18-month measurement (t(53.33) = −1.27, *p*-Value = 0.20) (Table 2).

#### 3.2.2. Sum of Curves Results

The analysis results showed a statistically significant effect of the “measurement time” factor (F(1.27) = 274.83, *p*-Value < 0.001, effect size = 1.88, power = 1) and a statistically significant interaction between the time and group factors (F(1.27) = 143.12, *p*-Value < 0.001, effect size = 1.36, power = 1). However, post-hoc tests did not reveal statistically significant differences between the groups at the 12-month measurement (t(78) = −1.87, *p*-Value = 0.065) or at the 6-month follow-up (t(78) = −1.94, *p*-Value = 0.56) (Table 2).

#### 3.2.3. ATR Maximum Results

The analysis results showed a statistically significant effect of the measurement time factor (F(1.31) = 58.51, *p*-Value < 0.001, effect size = 0.87, power = 1) and a statistically significant interaction between the time and group factors (F(1.31) = 42.56, *p*-Value < 0.001, effect size = 0.74, power = 1). Post-hoc tests revealed statistically significant differences between the groups at the 12-month measurement (t(77.99) = −7.44, *p*-Value < 0.001), with the intervention group showing a statistically significant improvement compared to the control group (mean difference −3.05). This difference remained statistically significant at the 18-month measurement (t(77.89) = −7.86, *p*-Value < 0.001) (Table 2).

#### 3.2.4. SRS-22 Results

The analysis results showed a statistically significant effect of the measurement time factor (F(1.42) = 29.66, *p*-Value < 0.001, effect size = 0.68, power = 1) and a statistically significant interaction between the time and group factors (F(1.42) = 26.43, *p*-Value < 0.001, effect size = 0.58, power = 1). Post-hoc tests revealed statistically significant differences between the groups at the 12-month measurement (t(59.17) = −6.58, *p*-Value < 0.001), with the intervention group showing a statistically significant improvement compared to the control group (mean difference −0.87). This difference remained statistically significant at the 18-month measurement (t(54.85) = −5.77, *p*-Value < 0.001) (Table 2).

**Table 2:** Mean values and standard deviations (M ± SD) of each variable (with upper and lower confidence interval limits) at each measurement time point, with *p*-Values for between groups and interaction effects (and Cohen’s d effect size).

Variable	Intervention Group M ± SD	Control Group M ± SD	Mean Difference Between Groups (95% CI)	Between Groups *p*-Values	Interaction *p*-Value/Effect Size (Cohen’s d)
Cobb Angle (degrees)					
Baseline Measurement	33.43 ± 5.98	34.65 ± 3.08	−1.22 (−3.34, 0.89)	0.25	*p*-Value < 0.001/Cohen’s d = 0.30
12-Month Measurement	27.80 ± 6.20	31.48 ± 2.66	−3.65 (−5.81, −1.53)	0.001 *	
18-Month Measurement	29.10 ± 5.69	30.35 ± 2.48	−1.25 (−3.22, 0.72)	0.20	
Sum of Curves (degrees)					
Baseline Measurement	53.23 ± 12.64	53.48 ± 14.40	−0.25 (−6.28, 5.78)	0.93	*p*-Value < 0.001/Cohen’s d = 1.36
12-Month Measurement	43.03 ± 10.96	48.23 ± 13.70	−5.20 (−10.72, 0.32)	0.65	
18-Month Measurement	44.63 ± 10.93	50.06 ± 13.86	−5.42 (−10.98, 0.13)	0.56	
ATR Maximum (degrees)					
Baseline Measurement	13.35 ± 2.93	13.08 ± 1.50	0.27 (−0.76, 1.31)	0.59	*p*-Value < 0.001/Cohen’s d = 0.74
12-Month Measurement	7.20 ± 1.84	10.25 ± 1.82	−3.05 (−3.86, −2.23)	<0.001 *	
18-Month Measurement	6.85 ± 2.27	10.77 ± 2.19	−3.92 (−4.91, −2.93)	<0.001 *	
SRS-22 (score)					
Baseline Measurement	3.27 ± 0.73	3.22 ± 0.77	0.04 (−0.28, 0.38)	0.77	*p*-Value < 0.001/Cohen’s d = 0.58
12-Month Measurement	4.33 ± 0.39	3.46 ± 0.74	0.87 (0.60, 1.13)	<0.001 *	
18-Month Measurement	4.39 ± 0.33	3.66 ± 0.72	0.72 (0.47, 0.97)	<0.001 *	

* indicates statistical significance at *p*-Value < 0.05.

## 4. Discussion

The purpose of our clinical study was to examine the impact of a long-term Schroth exercise program on people with AIS, specifically on the severity of their scoliosis and their quality of life while undergoing brace treatment.

The results of our study showed that the application of a long-term supervised Schroth exercise program significantly improved both the severity of scoliosis and the quality of life of participants in the intervention group compared to the control group, which received only the brace.

Regarding the Cobb angle, the analysis results showed a statistically significant improvement in the intervention group compared to the control group at the 12-month measurement. The mean Cobb angle in the intervention group decreased by 5.63° (16.84% reduction) from baseline, whereas the corresponding reduction in the control group was 3.17° (9.14% reduction). This greater improvement in the intervention group can be attributed to the long-term application of Schroth exercises, which helped in both halting the progression of spinal deformity and reducing the size of the primary curvature. Park et al. [14] noted that long-term Schroth interventions appear to be more effective, as their meta-analysis identified medium effect sizes for interventions shorter than 6 months and large effect sizes for programs longer than 6 months.

However, based on recent studies, the duration of Schroth programs varies from a few weeks to 6 months [10,14,15,24]. The implementation of a 12-month supervised Schroth exercise program in our study was innovative, as previous studies with long-term Schroth protocols typically transition to home exercises after a few weeks of supervised sessions [16,17]. Furthermore, the high participation rate in our study, even 12 months after its initiation, demonstrated that a long-term supervised Schroth program is feasible and can be combined with brace treatment as part of the long-term conservative management of AIS.

It is also important to note that although Cohen’s d effect size was low (Cohen’s d = 0.30) [21], the improvement observed in the intervention group exceeded 5°, which is considered the minimum clinically significant difference in individuals with AIS [15,25]. Therefore, the improvement in the Cobb angle is clinically significant as well.

Our findings align with those of Kwan et al. [16], who also found a statistically significant improvement in the Cobb angle after applying Schroth exercises in individuals with AIS who wore a brace, using a long-term Schroth intervention lasting more than 6 months.

However, it is important to mention that the statistical difference between the groups was not maintained in the 18-month measurement, which may be due to a partial worsening of the Cobb angle in the intervention group after the end of the 12-month program. This could be attributed to the low mean Risser index in the participants, indicating that their scoliosis still had the potential to worsen. Nevertheless, even though the difference in mean Cobb angle in the 18-month measurement was not statistically significant, the angle in the intervention group remained numerically improved compared to the control group (Table 2).

Another important aspect to highlight is that the severity of scoliosis among participants in this study was greater, as indicated by the mean Cobb angle (34.04 degrees), compared to other studies that have applied Schroth protocols in individuals with AIS [22,24]. Recent studies support the efficacy of Schroth exercises in individuals with AIS and smaller scoliosis (up to 25°) [12], whereas the effectiveness of the method in severe scoliosis is debated by some researchers [15].

Similar results were observed for the sum of curvatures with the intervention group improving twice as much as the control group in the 12-month measurement. Specifically, the improvement in the intervention group was 10.20° (19.16% improvement), whereas the corresponding improvement in the control group was 5.25° (9.81% improvement). However, post-hoc tests did not reveal statistically significant differences between the groups at any measurement time, with *p*-Values being marginally non-significant (*p*-Value = 0.65 in the 12-month measurement and *p*-Value = 0.56 in the 18-month measurement) (Table 2). A possible reason is the relatively small sample size and the large variability in the sum of curvatures, which prevented the detection of statistically significant differences despite the large numerical difference. Therefore, our study does not provide evidence that Schroth exercises further improved the sum of curvatures compared to brace-only treatment. Our results contradict those of Schreiber et al. [26], who found a statistically significant improvement in the sum of curvatures after a 6-month Schroth exercise program.

Regarding ATR maximum, the results showed that participants in the intervention group achieved greater improvement compared to the control group, with a statistically significant difference in the 12-month measurement. The mean ATR maximum decreased by 6.15° (46.06% improvement) from baseline, whereas the corresponding reduction in the control group was 2.83° (21.63% improvement). The greater improvement in the intervention group is logical, as similar results were observed for the Cobb angle of the primary curvature in the 12-month measurement and can be attributed to the positive effect of Schroth exercises. Additionally, the improvement in ATR maximum is clinically significant, as Cohen’s d effect size was moderate to high [21] (Cohen’s d = 0.74) (Table 2).

Moreover, it is important to mention that the statistically significant difference between the groups was maintained in the 18-month measurement, indicating long-term effects of the Schroth program on ATR maximum, which was not the case for the Cobb angle. The divergence in results between Cobb angle and ATR maximum (between the two groups) in the 18-month measurement was notable. However, research data support that the correlation between Cobb angle and ATR maximum in adolescents (10–18 years) with scoliosis is moderate, as it is influenced by other factors such as curvature type, age, and the way the musculoskeletal system compensates for scoliosis [27].

The results of this study are consistent with those of similar studies that also found improvement in ATR after applying Schroth exercises in individuals with AIS [16,20].

Regarding the SRS-22 score, the results showed a statistically significant improvement in the intervention group compared to the control group in the 12-month measurement. Specifically, the mean SRS-22 score increased by 1.06 points from baseline (21.20% improvement) in the intervention group, whereas the corresponding increase in the control group was 0.24 points (4.8% improvement), which indicates the positive impact of Schroth exercises on quality of life of the intervention group participants. Additionally, the statistically significant differences between the groups were maintained in the 18-month measurement, indicating that the positive impact of the Schroth program on the participants’ quality of life was sustained 6 months after the intervention. The improvement in the SRS-22 score in the intervention group is also clinically significant as the effect size was moderate (Cohen’s d = 0.58) [21]. The results of our study align with those of Kwan et al. [16], who also found a statistically significant improvement in the SRS-22 score with the addition of a long-term Schroth exercise program in individuals with AIS who wore a brace.

Our study faced several limitations that should be mentioned. The relatively small sample size and the fact that the sample was drawn from only one region of Greece may have affected the representativeness of the sample concerning the general population. Future research should aim to validate these findings with larger and more diverse samples, as well as explore the effects of different Schroth program durations in various populations with AIS.

## 5. Conclusions

Our study evaluated the impact of a long-term supervised Schroth exercise program on individuals with AIS who wear a brace. The results demonstrated that implementing a 12-month supervised Schroth exercise program is feasible and can lead to statistically and clinically significant improvements in both the severity of scoliosis and the quality of life for individuals with AIS. The study found a statistically significant improvement in the Cobb angle after completing the Schroth exercise program with the improvement exceeding the minimum detectable clinical difference of 5°. Additionally, a significant improvement in the ATR maximum was observed after the program, which was sustained 6 months post-intervention. Finally, the Schroth program had a significant impact on the SRS-22 score, which also remained improved 6 months after the intervention. Many of the positive effects showed were more pronounced compared to other studies that applied shorter-duration Schroth programs, suggesting that the effects of Schroth exercises may follow a linear dose–response relationship. Therefore, future clinical practice should consider implementing longer-duration Schroth programs. Further research is needed to provide more evidence on the efficacy of long-term Schroth exercise programs in individuals with AIS.

## Figures and Tables

**Table 1:** Demographic characteristics of participants by group and *p*-Values for group differences.

Demographic Characteristics of Participants	Intervention Group	Control Group	*p*-Values
Age (years) Mean (SD)	13.67 (1.19)	13.55 (1.02)	0.53
Gender (Male/Female) Frequency (%)	10% (*n* = 4) Male 90% (*n* = 36) Female	10% (*n* = 4) Male 90% (*n* = 36) Female	0.72
Risser Index (0–3) Mean (SD)	1.53 (1.01)	1.58 (0.98)	0.82
Age of Menarche (years) Mean (SD)	12.88 (1.03)	12.61 (0.95)	0.25
Cobb Angle of Primary Curvature (degrees) Mean (SD)	33.22 (6.27)	34.62 (3.14)	0.25
ATR max (degrees) Mean (SD)	13.58 (2.98)	13.08 (1.57)	0.60
Age at Diagnosis (years) Mean (SD)	10.54 (1.77)	11.17 (1.67)	0.15
Daily Brace Wear Time (hours) Mean (SD)	18.6 (1.97)	17.95 (1.88)	0.82
BMI (kg/m^2^) Mean (SD)	20.05 (2.45)	19.71 (2.02)	0.51
Family History (Yes/No) Frequency (%)	40% (*n* = 16) Yes 60% (*n* = 24) No	40% (*n* = 16) Yes 60% (*n* = 24) No	0.64
Regular Physical Activity (Yes/No) Frequency (%)	77.5% (*n* = 31) Yes 22.5% (*n* = 9) No	70.0% (*n* = 30) Yes 30.0% (*n* = 10) No	0.79

Mean (SD) = Mean (Standard Deviation).

## Data Availability

The datasets generated and analyzed during the current study are available from the corresponding author upon reasonable request.
